# Role of Inactive and Active *Trypanosoma cruzi Trans*-sialidases on T Cell Homing and Secretion of Inflammatory Cytokines

**DOI:** 10.3389/fmicb.2017.01307

**Published:** 2017-07-11

**Authors:** Leonardo Freire-de-Lima, Luciana B. Gentile, Leonardo M. da Fonseca, Kelli M. da Costa, Jessica Santos Lemos, Lucas Rodrigues Jacques, Alexandre Morrot, Célio G. Freire-de-Lima, Marise P. Nunes, Christina M. Takiya, Jose O. Previato, Lucia Mendonça-Previato

**Affiliations:** ^1^Laboratório de Glicobiologia, Instituto de Biofísica, Centro de Ciência da Saúde, Universidade Federal do Rio de Janeiro Rio de Janeiro, Brazil; ^2^Instituto Oswaldo Cruz, Fundação Oswaldo Cruz Rio de Janeiro, Brazil; ^3^Instituto de Microbiologia, Centro de Ciência da Saúde – Sala D1-035, Universidade Federal do Rio de Janeiro Rio de Janeiro, Brazil

**Keywords:** *Trypanosoma cruzi*, *trans*-sialidase, T cells, sialic acid, cytokines

## Abstract

*Trans*-sialidase from *Trypanosoma cruzi* (Tc-TS) belongs to a superfamily of proteins that may have enzymatic activity. While enzymatically active members (Tc-aTS) are able to transfer sialic acid from the host cell sialyl-glycoconjugates onto the parasite or to other molecules on the host cell surface, the inactive members (Tc-iTS) are characterized by their lectinic properties. Over the last 10 years, several papers demonstrated that, individually, Tc-aTS or Tc-iTS is able to modulate several biological events. Since the genes encoding Tc-iTS and Tc-aTS are present in the same copy number, and both proteins portray similar substrate-specificities as well, it would be plausible to speculate that such molecules may compete for the same sialyl-glycan structures and govern numerous immunobiological phenomena. However, their combined effect has never been evaluated in the course of an acute infection. In this study, we investigated the ability of both proteins to modulate the production of inflammatory signals, as well as the homing of T cells to the cardiac tissue of infected mice, events that usually occur during the acute phase of *T. cruzi* infection. The results showed that the intravenous administration of Tc-iTS, but not Tc-aTS protected the cardiac tissue from injury caused by reduced traffic of inflammatory cells. In addition, the ability of Tc-aTS to modulate the production of inflammatory cytokines was attenuated and/or compromised when Tc-iTS was co-injected in the same proportions. These results suggest that although both proteins present structural similarities and compete for the same sialyl-glycan epitopes, they might present distinct immunomodulatory properties on T cells following *T. cruzi* infection.

## Introduction

Although it has been discovered more than a 100 years ago, the Chagas disease is still an important public health problem, affecting millions of people in Latin American countries, and more than a million patients in Brazil only (Dias et al., 2016). The estimated cost of corrective surgeries and peacemaker implants derived from the cardiac chronic lesions can add up to 750 million dollars per year (Moncayo and Silveira, 2009).

Chagas disease is considered one of the most prevalent neglected tropical diseases, and its etiological agent is the protozoan parasite *Trypanosoma cruzi*, transmitted to vertebrate hosts by sucking triatomine insects, such as *Triatoma infestans, T. dimidiata*, and *Rhodnius prolixus* (Watanabe Costa et al., 2016). After infection, the infective forms invade a wide range of nucleated mammalian cells, including myocardial cells, promoting severe myocarditis that may kill up to 5% of untreated patients (Watanabe Costa et al., 2016). An ample spectrum of clinical presentations of the disease has been observed in humans, which may be attributed to the divergences among *T. cruzi* isolates and/or genetic differences in the host’s immune response (Brener, 1980; Lazzari et al., 2013).

Different *T. cruzi* strains, as well as the evolutionary forms of the parasite express different molecules on their surface (de Souza et al., 2010; Barrias et al., 2013). These cell surface molecules may be therapeutic targets, since most of them interact with host components to invade mammalian cells. Among these molecular targets are the enzymes denominated *trans*-sialidases (Tc-TS). Tc-TS belongs to a multigene family described over than 25 years ago, which encodes enzymatically active (aTc-TS) and inactive (iTc-TS) members (Cremona et al., 1999). Even though these two proteins can be distinguished by the single Tyr342His mutation (Cremona et al., 1995), both show similar substrate-specificity for α-2,3-sialic acid (SIA) and β-galactosyl (β-Gal) residues. Although this naturally occurring Tyr342→His substitution completely abolishes the Tc-TS activity (Cremona et al., 1995), the inactive members are still able to modulate parasite-host cell interactions through their lectinic properties (Todeschini et al., 2002a,b, 2004).

Since *T. cruzi* is incapable of synthetizing SIA, it falls onto Tc-aTS to transfer terminal SIA from the host cell surface molecules to its own glycoconjugates (Previato et al., 1985), affecting invasion, host immune response, cell adhesion and cell signaling (Schenkman et al., 1993; Hinou et al., 2005; Carvalho et al., 2010). The genes encoding Tc-aTS and Tc-iTS exhibit the same number of copies (Egima et al., 1996; Cremona et al., 1999). Due to the shortage of specific antibodies, no studies have demonstrated the differential effect and/or expression of both proteins in the course of infection.

SIA levels in T cell surface are known to change in the early events of immune response (Galvan et al., 1998; Harrington et al., 2000). In an earlier study, our group has demonstrated that Tc-aTS is capable of resialylating cytotoxic CD8^+^ T cells, effectively dampening the host immune response against the parasite (Freire-de-Lima et al., 2010). In the present study, we intend to unveil the effects of Tc-aTs and Tc-iTS in the cardiac injury caused by *T. cruzi* infection, as well as their influence on host cell immunomodulation. Here we demonstrated, for the first time, the individual and combined immunobiological effects triggered by Tc-aTS and Tc-iTS in *T. cruzi*-acutely infected mice.

## Materials and Methods

### Purification of Recombinant Tc-aTS and Tc-iTS

Both recombinant proteins used in this study contained the C-terminal repeats and were obtained from *Escherichia coli* MC1061 electro-transformed with plasmids containing either the wild-type TS insert, TSREP, or the inactive mutant TS insert bearing a Tyr342 → His substitution, pTrcHisA (Cremona et al., 1995). Proteins were purified as described previously (Todeschini et al., 2000), and their homogeneity was evaluated by 10% SDS–PAGE. Prior to all experiments, Tc-aTS and Tc-iTS were eluted through an agarose-polymyxin B column (Sigma) in order to obtain lipopolysaccharide-free preparations. The lipopolysaccharide content of TS preparations was below the detection limit for the Limulus amebocyte lysate assay (Charles River Endosafe, Charleston, SC, United States).

### Animals and Infection

BALB/c mice (male, aging 6–8 weeks) were obtained from Fundação Oswaldo Cruz, Rio de Janeiro, Brazil. Bloodstream trypomastigotes of the Y strain were obtained from *T. cruzi*-infected mice 8 days post-infection (dpi). After adjustment of the parasite concentration, each mouse was inoculated intraperitoneally (i.p.) with 0.1 ml of medium containing 10^4^ blood trypomastigotes. Parasitemia was monitored by counting the number of trypomastigotes in blood samples from the tail-cuff of infected animals (Brener, 1962).

### *Trans*-Sialidase Treatment

In order to evaluate the effect of Tc-TS proteins on *T. cruzi*-infected (I) and non-infected animals (NI), BALB/c mice were divided in six experimental groups: (a) NI, (b) NI and treated with Tc-aTS (NI-aTS), (c) NI and treated with Tc-iTS (NI-iTS), (d) I, (e) I and treated with Tc-aTS (I-aTS), and (f) I and treated with Tc-iTS (I-iTS). The NI groups (NI-aTS and NI-iTS) were daily injected with 30 μg of Tc-aTS or Tc-iTS on days 1, 2, and 3 and scarified at 15 dpi. The I groups (I-iTS and I-aTS) were treated as described above, but received the first dose of recombinant Tc-TS proteins 1 h before infection. Untreated controls received only phosphate buffered saline (PBS). In order to investigate a possible neutralizing effect of Tc-iTS on Tc-aTS, *T. cruzi*-infected mice were injected with equal amount of Tc-TS proteins (30 μg) as well as an additional point where Tc-iTS amounts were three times higher than Tc-aTS (30:90 μg), and the effect on inflammatory cytokines secreted by splenic T cells was analyzed.

### Histopathology

The hearts were surgically removed, embedded in tissue freezing medium (Tissue Tek OCT, United States) as described (dos Santos et al., 2001), sectioned with a cryostat in 6 μm thick sections, placed on poly-L-lysine-containing slides and fixed for 20 min in buffered formalin pH 7.2. The slides were mounted on glass slides, stained with hematoxylin and eosin (HE) and photomicrographs were taken at 40× objective lens under a light field microscope (Eclipse E800, Nikon, Japan) coupled to a digital camera (Evolution Media Cybernetics Inc., Bethesda, MD, United States) for the evaluation of the inflammatory cell infiltrate and the number of parasite nests per field (dos Santos et al., 2001; Mello et al., 2015). At least 30 histological fields from four different animals were randomly selected out of each experimental group (1 field = 0.159 mm^2^), and high-quality images (2,048 × 1,536 pixel buffer) were captured after setting and calibrating the program. The images were analyzed using Image Pro Plus 4.5.1 software (Media Cybernetics).

### Immunohistochemistry

The hearts were surgically removed, embedded in tissue freezing medium (Tissue Tek, OCT USA) as previously described (dos Santos et al., 2001), sectioned using a cryostat into 6 μm sections, placed on slides containing poly-L-lysine and fixed with acetone for 20 min. Subsequently, the sections were washed with saline and blocked with Fc receptor blocker for 20 min. The slides were incubated overnight with primary monoclonal antibodies directed against T cell markers. The primary antibodies used were purified rat anti-mouse CD4 and purified rat anti-mouse CD8 (Pharmingen, United States). Subsequently, the slides were washed with PBS-Tween 0.25% (PBS/T), and incubated with biotinylated goat anti-rat IgG antibody (Vector Laboratories, United States) for 60 min. After that, the slides were washed for 10 min with PBS and incubated for 45 min with streptavidin, horseradish peroxidase (HRP) (Vector Laboratories), followed by washing with PBS for 10 min. The antigen detection was performed using DAB (3,3′ diaminobenzidine) Peroxidase Substrate Kit (Vector Laboratories, United States). At least 30 fields from four different animals were analyzed in each group (1 field = 0.159 mm^2^). The tissues were counterstained with haematoxylin. Positive controls consisted of known positive histological sections and, as negative control, histological fragments were processed normally, but the primary antibody was omitted, and substituted by its isotype controls (Pharmingen, United States). Immunohistochemical sections were evaluated under a light microscope (Eclipse E800, Nikon, Japan) coupled to a digital camera (Evolution Media Cybernetics Inc., Bethesda, MD, United States), and photos were taken at 40× objective lens. The images were analyzed using Image Pro Plus 4.5.1 software (Media Cybernetics). Photomicrographs were subjected to manual counting of marked cells (Carvalho et al., 2012; Pereira et al., 2014).

### Creatine Kinase MB (CK-MB) Activity

The creatine kinase MB (CK-MB) activity, which is the cardiac isoform of CK, was measured as described (de Souza et al., 2000; Henriques-Pons et al., 2002). Briefly, heparinized plasma samples from the six experimental groups were collected at 15 dpi. This assay was used as a marker for myocardial injury, and the results are shown as the rate of increase in NADPH (Delta E/min) after seven consecutive readings at 1 min intervals at 340 nm in a multiwheel spectrophotometer (BioTek Instruments, United States).

### Isolation of Cardiac Inflammatory Cells

Hearts extracted from mice of the six experimental groups were cut into fragments from 1 to 2 mm thick in ice-cold PBS. The fragments were then transferred to a 0.2% solution of type IV collagenase (5.2 U/mg) (Sigma–Aldrich, St. Louis, MO, United States) and submitted to four or five 20-min enzymatic digestion cycles under gentle agitation at 37°C. The isolated cells were centrifuged and kept on ice-cold RPMI 1640 medium supplemented with 10% FBS and maintained in ice (Cascabulho et al., 2012).

### Total DNA Extraction and Real Time PCR Analysis

Total genomic DNA from cardiac tissue of non-infected and *T. cruzi*-infected mice treated or not with recombinant Tc-aTS or Tc-iTS was extracted through the digestion of thin tissue slices (∼20 mg). Digestion was performed with 150 μl of lysis buffer (25 mM NaOH, 0.2 mM Na2-EDTA) after heating at 95°C for 1 h. Subsequently, the material was cooled for 20 min at 4°C and 150 μl of the neutralization buffer (40 mM Tris-HCl) was added to each sample. The samples were centrifuged at 8500 ×*g* for 20 min, and total DNA from the supernatant was quantified in a GeneQuant RNA/DNA Calculator spectrophotometer (Biochrom, United Kingdom). The assay was performed on 96-well plates (Applied Biosystems, United Kingdom) and processed by ABI Prism 7900 Sequence Detection System (Applied Biosystems, United Kingdom). Reactions were performed in a final volume of 25 μl containing: total DNA (0.1 μg), SYBR, 3 μl of 25 mM MgCl_2_; 2 μl of 10 mM dNTP, 0.1 μl of AmpliTaq Gold^®^ 5 U/μl, and 1 μl of the forward and reverse primers, specific for *T. cruzi* mini-circles. The following primers were used for the forward and reverse strands, respectively: GCTCTTGCCCACAMGGGTGC, where M = A or C and CCAAGCAGCGGATAGTTCAG (Cummings and Tarleton, 2003).

### Flow Cytometry

For flow cytometry analysis, we digested the heart tissue as described above and after centrifugation, all obtained cells were resuspended in RPMI 1640 medium supplemented with 10% FBS and 10% inactivated normal sheep serum and incubated for 30 min at 4°C for FcγR blockage. Afterward, cells were counted on a hemocytometer and incubated with previously titrated APC/Cy7-conjugated anti-CD8 and PercP-conjugated anti-CD4 monoclonal antibodies (all from eBioscience, San Diego, CA, United States) for 30 min in ice, washed twice in RPMI 1640 medium and fixed using 2% formaldehyde (EMD Chemicals, Gibbstown, NJ, United States) in PBS until acquisition in a FACSCalibur flow cytometer (BD Biosciences, San Jose, CA, United States). Ten thousand events were acquired for all analyzed samples. Data analysis was performed using Summit 4.3 software from Dako (Cascabulho et al., 2012).

### T Cell Purification

Cell populations enriched for T lymphocytes from mice of the six experimental groups were obtained by nylon wool filtration of unfractionated splenic cell suspensions as previously described (Freire-de-Lima et al., 2000). T cells were cultured in DMEM supplemented with 2 mM glutamine, 5 × 10^-5^ M 2-Mercaptoethanol (2-ME), 10 μg/mL gentamicin, 1 mM sodium pyruvate, and 0.1 mM MEM non-essential amino acids (all from Gibco^TM^, Invitrogen Corporation) plus 1% Nutridoma-SP (Roche, Germany) instead of FBS (Nunes et al., 2013).

### Elisa for Detection of Inflammatory Cytokines Secreted by Splenic T Cells

Ninety-six-well plates (Nunc, United States) were coated with 100 μl/well of capture antibodies at 4 μg/mL (Santa Cruz Biotechnology, United States) and incubated overnight at 4°C. The plates were washed twice with PBS/T, and blocked with PBS supplemented with 10% FBS (PBS/10% FBS) in a volume of 200 μl/well. Plates were allowed to stand for 2 h at 37°C and washed twice with PBS/T. Subsequently, the standards were diluted in PBS/10% FBS and added to the wells. The supernatants from the splenic T cells cultures from each experimental group were collected 48 h after the purification procedure, and 50 μl of each were added to the coated plates (Freire-de-Lima et al., 2000). The plates were incubated overnight at 4°C. Afterward, the plates were washed four times with PBS/T and 4 μg/mL of detection antibodies (Santa Cruz Biotechnology, United States) were added to the wells. The plates were incubated for 1 h at room temperature and washed six times with PBS/T. Subsequently, 100 μl of streptavidin phosphatase (1 μg/mL) diluted in PBS/10% FBS were added to the wells. The plates were incubated for 3 h at room temperature. Then, the plates were washed eight times with PBS/T and 1 mg/mL solution of bis-azine ethyl benzothiazole sulfonic acid substrate (Sigma, United States) and 100 mM MgCl_2_ diluted in 20 mM Tris was added to the wells. The readings were performed in a Beckman Coulter AD 340 reader with a 405 nm filter.

### Statistical Analysis

Statistical analyses were performed with GraphPad Prism 5 software. Statistical differences between mean values were evaluated by one-way analysis of variance (ANOVA), followed by Tukey’s Multiple Comparison Test. Results were expressed as mean ± standard deviation (SD), and differences between control and treated group were considered statistically significant when *p* ≤ 0.05.

### Ethics Statement

The experiments were carried out in strict accordance with the recommendations in the Guide for the Care and Use of Laboratory Animals of the National Institutes of Health (United States). The protocol was approved by the Committee on the Ethics of Animal Experiments of the Health Science Center of the Federal University of Rio de Janeiro (CEUA-CCS, Permit Number: IBCCF 062/14), and all efforts were made to minimize suffering.

## Results

Current literature shows that the main diseases and conditions associated with Chagas disease are cardiac complications, such as conduction disorders/arrhythmias (41.4%) and heart failure (37.7%) (Martins-Melo et al., 2012). Since our previous work (Freire-de-Lima et al., 2010) showed that intravenous administration of Tc-TS proteins influence the survival of *T. cruzi*-infected mice, we have decided to analyze the frozen tissues obtained from the animals used in our previous study (Freire-de-Lima et al., 2010) to verify the inflammatory infiltrate in the cardiac tissue.

**FIGURE 1:**
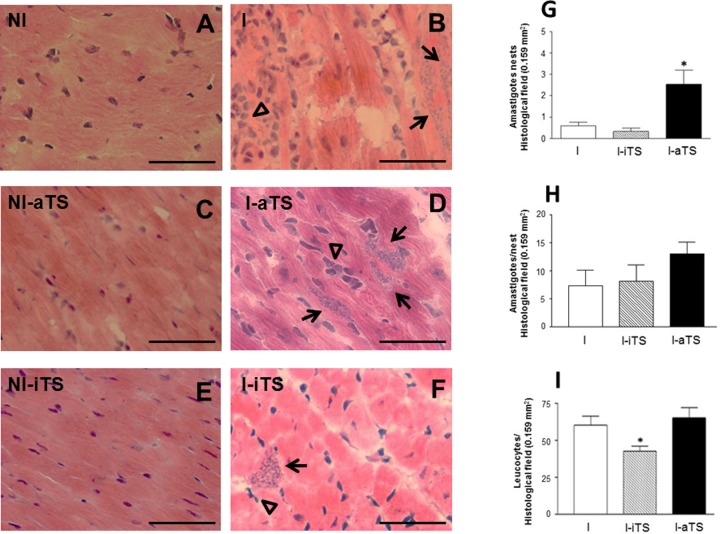
*Trypanosoma cruzi*-infected mice treated with recombinant Tc-aTS or Tc-iTS present an increase in the number of amastigote nests and reduced number of infiltrating leukocytes in the cardiac tissue. Balb/c mice were infected and treated or not with Tc-iTS or Tc-aTS. Histopathological analysis of the heart tissue from all experimental groups: NI **(A)**, I **(B)**, NI-aTS **(C)**, I-aTS **(D)**, NI-iTS **(E)**, and I-iTS **(F)** were performed at 15 days post-infection (dpi). Photomicrographs were taken at 40× objective lens under a light field microscope coupled to a digital camera for the evaluation of the inflammatory cell infiltrate and the number of parasite nests per field. At least 30fields of four different animals were randomly selected (1 field = 0.159 mm^2^), nd high-quality images were captured. The images were analyzed using Image Pro Plus 4.5.1 software (Media Cybernetics). Graphs with error bars represent the mean ± SD of the number of amastigote nests (**G**, arrows, ^∗^*p* ≤ 0.05 versus I and I-iTS groups), the number of amastigotes per nest **(H)** and infiltrating leukocytes (**I**, arrowheads, ^∗^*p* ≤ 0.05 versus I and I-aTS groups) in the cardiac tissue of *T. cruzi*-infected mice. Bars: 50 μm, *n* = 7 animals per group.

Histopathological studies revealed that the groups NI-aTS and NI-iTS did not show alteration in the number of infiltrating leukocytes when compared to the non-infected control group. In these animals, the cardiac tissue presented its typical characteristic, with the presence of rare infiltrating leukocytes (**Figures 1A,C,E**). However, in the cardiac tissue of infected animals (**Figures 1B,D,F**) it was possible to identify striking differences. A significant increase in the number of amastigote nests in the I-aTS group (**Figure 1D**, arrows and **Figure 1G**, black bar) was evidenced when compared to the I and I-iTS groups (**Figures 1B,F**, arrows and **Figure 1G**, white and scratched bars). However, no difference in the number of amastigotes per nest was observed between the groups (**Figure 1H**). Results obtained by qPCR corroborated the histopathological analysis, since the concentration of *T. cruzi* DNA detected in the cardiac tissue of I-aTS group was ∼sevenfold greater than what was found in the cardiac tissue of I and I-iTS groups (**Supplementary Figure S1**). In addition, the quantification of the number of leukocytes revealed that the administration of Tc-iTS markedly reduced the number of infiltrating leukocytes in the heart (**Figure 1F**, arrowhead and **Figure 1I**, scratched bar) when compared to the I and I-aTS groups (**Figures 1B,D**, arrowheads and **Figure 1I**, white and black bars). This result might be explained by the lectin property of Tc-iTS (Todeschini et al., 2004). In addition, Dias et al. (2008), demonstrated that Tc-iTS binds sialyl-glycan epitopes on endothelial cell surface. That event is capable of both inducing the activation of the NF-kappaB pathway, and the expression of the adhesion molecules E-selectin, ICAM-1 and VCAM-1, resulting in an increase of parasite invasion into host cells (Dias et al., 2008), further pointing out the importance of Tc-TS lectinic property as a virulence factor. It is plausible to inquire whether Tc-iTS might be associated to α2–3 SIA and β-Gal*p* units present in structures such as the sialyl lewis epitope, which besides being a component of biologically important glycoconjugates such as LFA-1, CD62-L, and CD43, acts as an E-selectin ligand (Kannagi, 2002).

**FIGURE 2:**
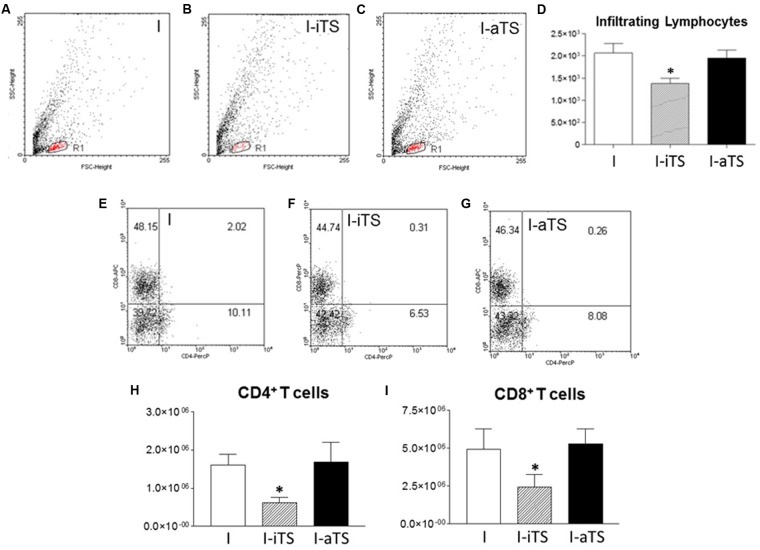
Intravenous administration of recombinant Tc-iTS reduces the infiltration of both T cell subsets in the heart of *T. cruzi*-infected mice. Balb/c mice were infected and treated or not with Tc-iTS or Tc-aTS. After 15 days, mice were euthanized, and the heart were digested in a solution containing 0.2% collagenase IV. Cell homogenates were labeled with fluorescent antibodies specific for CD4^+^ and CD8^+^ T cells, and analyzed by flow cytometry. **(A–C)** Dot plots represent the lymphocyte region (R1) relative to the I, I-iTS, and I-aTS groups. **(D)** Bar graph shows the absolute number of infiltrating lymphocytes. **(E–G)** Dot plots show the relative number of CD4^+^ and CD8^+^ T cells. **(H,I)** Bar graphs show the absolute number of infiltrating CD4^+^ and CD8^+^ T cells. (^∗^*p* ≤ 0.05 versus I and I-aTS groups). Error bars are mean ± SD, *n* = 7 animals per group.

The reduction in the number of infiltrating leukocytes in the I-iTS group was also observed during the flow cytometry analysis. **Figures 2A–C** show a clear difference in the number of infiltrating cells in the dot plot region corresponding to lymphocytes (R1). This can also be seen in **Figure 2D**, which represents the average of gated cells in the dot plots (R1 region) of seven individual experiments. No change was observed between NI groups (data not shown). Despite this reduction, there is no difference between the percentages of CD4^+^ and CD8^+^ T cells of the NI (data not shown) and I groups (**Figures 2E–G**). It is important to notice that absolute number of both T cell subsets was reduced in the I-iTS group (**Figures 2H,I**, scratched bars).

**FIGURE 3:**
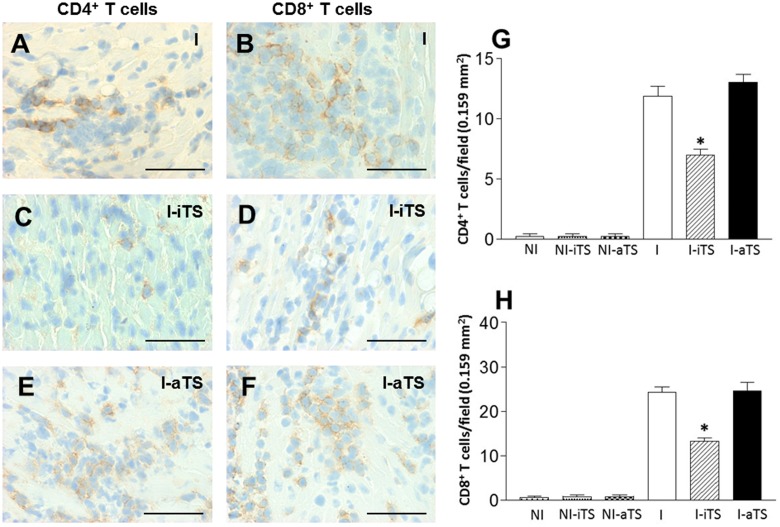
Intravenous administration of Tc-iTS compromises the trafficking of CD4^+^ and CD8^+^ T cells to the heart of *T. cruzi*-infected mice. Balb/c mice were infected and treated or not with Tc-iTS or Tc-aTS. After 15 days, mice were euthanized, and the hearts were subjected to immunohistochemistry analysis for detection of CD4^+^ **(A,C,E)** and CD8^+^ **(B,D,F)** infiltrating T cells. Immunohistochemical sections were evaluated under a light microscope coupled to a digital camera, and photos were taken at 40× objective lens. At least thirty fields of four different animals were randomly selected (1 field = 0.159 mm^2^), and high-quality images were captured. The images were analyzed using Image Pro Plus 4.5.1 software (Media Cybernetics). **(G,H)** Graphs with error bars represent the mean ± SD of CD4^+^ and CD8^+^ T cells in 100 microscopic fields (^∗^*p* ≤ 0.05 versus I and I-aTS groups). Bars: 50 μm, *n* = 7 animals per group.

In order to better understand the effect of Tc-TS in the migration of T cells into cardiac tissue, non-infected and infected BALB/c mice were treated with Tc-iTS and Tc-aTS, and the degree of infiltration of both T cell subsets was assessed by immunohistochemistry. The results demonstrated that the cardiac tissue of the I-iTS group (**Figures 3C,D,G,H**, scratched bars) presented a marked reduction in the number of CD4^+^ and CD8^+^ T cells when compared to the I and I-aTS groups (**Figures 3A,B,E–H**).

**FIGURE 4:**
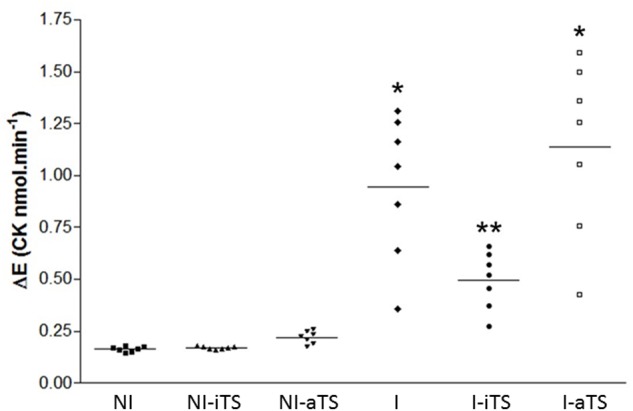
*Trypanosoma cruzi*-infected Balb/c mice show a decrease in plasma activity of creatine kinase (CK) after treatment with Tc-iTS. The analyzes obtained by flow cytometry and immunohistochemistry revealed that *T. cruzi*-infected mice treated with recombinant Tc-iTS showed a reduction in the number of inflammatory cells in the heart tissue when compared to I and I-aTS groups. To validate this hypothesis, at 15 dpi, mice were euthanized and the plasma used to determine the CK-MB activity. The results are expressed as the rate of increase in NADPH (Delta E/min) after seven sequential readings at 1 min intervals at 340 nm in a multiwheel spectrophotometer (BioTek Instruments). Error bars are mean ± SD. (^∗^*p* ≤ 0.01 versus NI, NI-iTS, NI-aTS, and I-iTS groups). (^∗∗^*p* ≤ 0.05 versus NI, NI-iTS, and NI-aTS groups), *n* = 6 animals per group.

To evaluate the degree of cardiac fiber injury, plasmatic CK activity was evaluated at 15 dpi. Corroborating the histopathological and immunohistochemical analysis, the I-iTS group presented a marked reduction of CK activity when compared to I and I-aTS groups. No difference in CK activity was observed between the NI groups (**Figure 4**).

**FIGURE 5:**
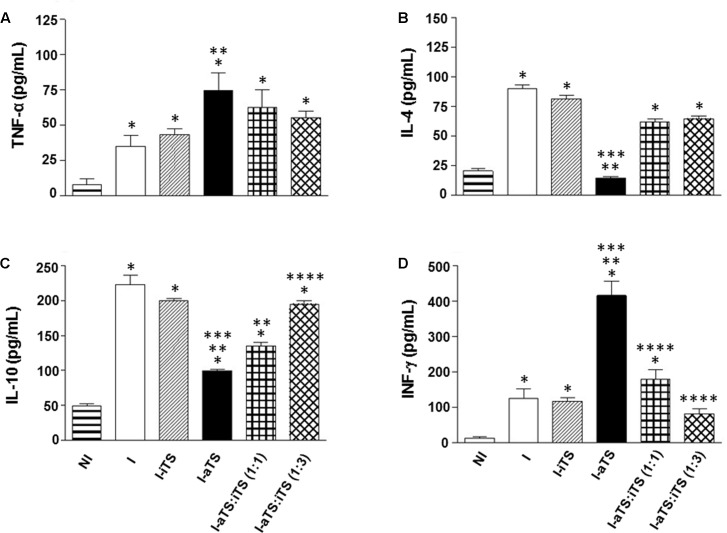
Tc-iTS reverses the secretion of inflammatory signals induced by Tc-aTS. Balb/c mice were infected and treated or not with Tc-iTS or Tc-aTS. In order to analyze a possible neutralizing effect of Tc-iTS on Tc-aTS, *T. cruzi*-infected mice were injected with equal amount of Tc-TS proteins, as well as an additional point where Tc-iTS amounts were three times higher than Tc-aTS. After 8 days, mice were euthanized and T cell-enriched populations were obtained by nylon wool filtration of unfractionated splenic cell suspensions. The secretion of inflammatory cytokines TNF-α **(A)**, IL-4 **(B)**, IL-10 **(C)**, and INF-γ **(D)** was accessed by ELISA. The readings were performed in a Beckman Coulter AD 340 reader with at 405 nm filter. Error bars are mean ± SD. (^∗^*p* ≤ 0.05 versus NI group, ^∗∗^*p* ≤ 0.05 versus I group, ^∗∗∗^*p* ≤ 0.05 versus I-iTS group, and ^∗∗∗∗^ *p* ≤ 0.05 versus I-aTS group), *n* = 6 animals per group.

It is well described that Tc-aTS e Tc-iTS show similar substrate-specificity for α-2,3-SIA and β-Gal residues (Cremona et al., 1999; Todeschini et al., 2004), indicating that both proteins might compete *in vivo* by the same ligands during the acute experimental Chagas disease, governing numerous immunobiological phenomena. In order to evaluate this possibility, infected BALB/c mice were injected with equal amount of Tc-TS proteins (30 μg) as well as three times more Tc-iTS than Tc-aTS (30:90 μg), and the spontaneous secretion of inflammatory cytokines (TNF-α, INF-γ, IL-4, and IL-10) by splenic T cells, as well as the number of blood trypomastigotes were evaluated. The results showed that when Tc-iTS was co-injected with the same amount or even with thrice the amount, the effect of Tc-aTS in the cytokine profile (**Figures 5A–D**), as well its ability to increase the blood peripheral parasitemia (**Supplementary Figure S2**) became attenuated or altogether abrogated. Taken together, these results demonstrate that the enzymatic activity displayed by Tc-TS proteins is essential for such phenomena.

## Discussion

Throughout human history, infectious agents have developed effective mechanisms to dampen the host immune response (Ernst, 2016). Since protozoans are considered the most ancient members within the animal kingdom, it is plausible to speculate that they developed sophisticated strategies to guarantee their survival as intracellular parasites (Nardy et al., 2016). *T. cruzi* is a good and clear example, since the parasite can make use and/or modify the host cell glycophenotype to their own benefits (Jackson, 2015; Mucci et al., 2017).

It is well established that *T. cruzi* expresses inactive and active TS proteins. The enzymatic activity of Tc-TS has been described as a virulence factor during *T. cruzi* infection, contributing for tissue damage and parasite persistency in the infected host (Chuenkova and Pereira, 1995; Leguizamon et al., 1999; Chuenkova et al., 2001; Mucci et al., 2002; Chuenkova and Pereira, 2003; Tribulatti et al., 2005; Risso et al., 2007; Freire-de-Lima et al., 2010; Muia et al., 2010; Mucci et al., 2017), while its inactive analog (Tc-iTS) presents a single point mutation, Tyr342 → His. The Tyr342 residue is involved in the stabilization of the sialyl carbocation transition state, formed during the hydrolysis reaction of the Tc-aTS, and the presence of His342 impairs enzymatic activity (Cremona et al., 1995). Previous work (Todeschini et al., 2002a, 2004) demonstrated that the recombinant Tc-iTS is a SIA-binding protein, displaying the same specificity required by Tc-aTS. It was demonstrated that Tc-iTS physically interacts with the SIA from the sialomucin CD43 on CD4^+^ T cells (Todeschini et al., 2002b).

A previous work from our group demonstrated that when administered intravenously into *T. cruzi*-infected mice, Tc-aTS and Tc-iTS have opposite effects on mice survival. The results showed that the experimental group injected with Tc-aTS succumb to disease significantly earlier when compared to the group that received Tc-iTS (Freire-de-Lima et al., 2010). However, the molecular mechanism responsible for such phenomenon is currently missing. Several papers have shown that following *T. cruzi* infection, Tc-TS proteins influence the architecture and distribution of cells in different organs and tissues (Mucci et al., 2002; Tribulatti et al., 2005; Mucci et al., 2006, 2017; Freire-de-Lima et al., 2015; Teixeira et al., 2015; Freire-de-Lima et al., 2016; Nardy et al., 2016; Savino, 2017). In particular, changes in the sialoglycophenotype of T lymphocytes seem to be an efficient mechanism adopted by the parasite to modulate the host immune response (Chuenkova and PereiraPerrin, 2004; Muia et al., 2010; Weinkauf et al., 2011; Freire-de-Lima et al., 2015, 2016; Lantos et al., 2016).

During development and activation, T cells undergo substantial changes in *O*- and *N*-linked glycan structures (Piller et al., 1988; Demetriou et al., 2001) and terminal addition of SIA to *O*- and *N*-glycans (Baum et al., 1996). Over the last 20 years, several works demonstrated that changes in SIA content are able to modulate numerous immunobiological phenomena in both mature and immature T cells (Hernandez et al., 2007; Drake et al., 2009; Earl et al., 2010; Redelinghuys et al., 2011; Rabinovich et al., 2012; Naito-Matsui et al., 2014). It is now well accepted that loss of cell surface SIA following T cell activation, enhances recognition of antigenic peptides bound to either class I or class II MHC surface molecules to initiate an adaptive immune response (Xu and Weiss, 2002; Bi and Baum, 2009). Since *T. cruzi* expresses an enzyme capable of modulate the sialoglycophenotype of mammalian host cells, the need to investigate how such changes may influence the behavior of CD8^+^ T cells becomes evident. This is especially urgent, since the immune response mediated by these cells is the most important against parasites residing in tissues, such as the cardiac muscle (Lannes-Vieira, 2003; Tarleton, 2015).

A previous work from our group demonstrated that following infection, splenic CD8^+^ T cells from *T. cruzi*-infected mice treated with recombinant Tc-aTS were positive for both activation marker CD44, as well as for *Maackia amurensis* (MAA), a lectin specific for α2-3 SIA, suggesting that Tc-aTS resialylates cell surface glycoconjugates of activated CD8^+^ T cells (Yamamoto et al., 1997; Freire-de-Lima et al., 2010). We demonstrated both *in vitro* and *in vivo* that although splenic CD8^+^ T cells exhibit an activated phenotype (CD44^high^), the sialoglycophenotype, modified by the action of Tc-TS activity, compromises the triggering of its cytotoxic activity, contributing to the increase of peripheral blood parasitemia (Freire-de-Lima et al., 2010). Based on these previous results from the splenic lymphocyte population, it is likely that heart-infiltrating CD8^+^ T cells would exhibit the same behavior. In the last 15 years, several papers described that in murine models of Chagas disease, an elevated peripheral blood parasitemia is associated with high cardiac tissue parasitism (Michailowsky et al., 2001; Waghabi et al., 2002; Cummings and Tarleton, 2004; Roffe et al., 2012). Such data corroborates the results initially described by Chuenkova and Pereira (1995), and our own histopathological findings, confirming that aTS treated mice exhibit higher parasitemia both in tissue and blood when infected with *T. cruzi*.

Several papers published by Campetella’s group demonstrated that administration of minute amounts of recombinant Tc-aTS in non-infected mice is capable of inducing apoptosis of mature and immature T cells (Leguizamon et al., 1999; Mucci et al., 2002, 2005, 2006), an event that naturally occurs following *T. cruzi* infection (DosReis and Lopes, 2009; Bienvenu et al., 2010; Decote-Ricardo et al., 2017). Since apoptosis induction is a mechanism commonly employed by *T. cruzi* to evade host immune responses (Freire-de-Lima et al., 2000; Lopes et al., 2000), the hypothesis that Tc-aTS-induced T cell death may be contributing to increased parasite burden cannot be ruled out. However, in *T. cruzi*-infected mice, we did not find any changes in the ratio of heart-infiltrating apoptotic T cells when Tc-aTS was administrated (data not shown). Additional studies are needed in order to clarify this question.

The results obtained by histopathological and immunohistochemical analysis demonstrated that the I-iTS group presented a reduced number of infiltrating leucocytes and T cells in the cardiac tissue, respectively. A study using endothelial cells derived from human bone marrow (HBMEC) and endothelial cells from porcine aorta (PAEC) demonstrated that Tc-iTS binds to α2,3-SIA containing molecules on those cells (Dias et al., 2008). Furthermore, Tc-iTS binds to α2,3-SIA present on the leucosialin CD43 expressed by T cells (Todeschini et al., 2002a). It is well known that glycans and glycan-binding proteins are important to the proper function of the immune system. Examples include the selectins, which are expressed in all types of leukocytes, and bind to endothelial cells in a glycan dependent event to ensure the extravasation of leukocytes into target tissues, an essential phenomenon for efficient control of infections (Daniels et al., 2002; Ley and Kansas, 2004; Mitoma et al., 2007; Marth and Grewal, 2008; Johnson et al., 2013). As lectin/carbohydrate recognition plays a major role in leukocyte/endothelial cell interaction, it is plausible to speculate that Tc-iTS might bind to SIA-containing adhesion molecules expressed in peripheral tissue homing receptors, impairing the homing of inflammatory cells to the heart tissue of *T. cruzi*-infected mice. Although Tc-iTS and Tc-aTS share a similar substrate specificity, an important and unresolved question is whether the sialylation or de-sialylation of cell surface glycoconjugates mediated by Tc-aTS might be able to alter the function of cell adhesion molecules. Since both addition and removal of SIA are dynamic phenomena, occurring continuously in the course of *T. cruzi* infection (Freire-de-Lima et al., 2015), it is plausible to consider that glycoconjugates involved in cell adhesion events are able to keep their functional properties, since many of those molecules carry SIA residues (Varki, 2007; Sperandio et al., 2009; Varki and Gagneux, 2012). It may explain why the administration of Tc-aTS does not alter the homing of T cells to the cardiac tissue of *T. cruzi*-infected mice. Additional experiments, such as adoptive transfer of labeled T cells from *T. cruzi*-infected mice upon injection of Tc-aTS *vs* Tc-iTS, may help us confirm the hypothesis about the differential regulation of T cell homing in response to both proteins. Although the arrival of CD8^+^ T cells to the cardiac tissue of *T. cruzi*-infected mice was not modulated by the administration of Tc-aTS, the parasite load was significantly higher as compared to the I and I-iTS groups. These results suggest that the functional properties of CD8^+^ T cells might be attenuated or compromised in the presence of Tc-aTS. Further studies are required to evaluate the cytotoxic effect, as well as the phenotypic characteristics of heart-infiltrating CD8^+^ T cells in response to Tc-TS activity.

It has been demonstrated that in endothelial cells and T lymphocytes, the expression of chemokine receptors may be modulated during *T. cruzi* infection (Lannes-Vieira, 2003; Talvani et al., 2004; Machado et al., 2005; Hao et al., 2017). Since their half-lives and functions are dependent of its glycosylation state (Ludwig et al., 2000; Huskens et al., 2007; Ostuni et al., 2014), it would be important to investigate if changes in the sialoglycophenotype of chemokine receptors are able to control the influx of inflammatory cells to the heart of *T. cruzi*-infected mice.

Previously, we demonstrated that the administration of recombinant Tc-iTS in *T. cruzi*-infected mice was not able to alter the peripheral blood parasitemia when compared to untreated controls. However, CD8^+^ T cells presented high positivity for *Peanut agglutinin* (PNA), which binds to terminal β-gal residues (Sharma et al., 1998), suggesting that Tc-iTS might bind to host sialyl-glycoconjugates, thereby inhibiting a natural re-sialylation event that takes place during the acute phase of infection (Freire-de-Lima et al., 2010). Since the reduced SIA content potentiates the effector functions of CD8^+^ T cells (Pappu and Shrikant, 2004; Sadighi Akha et al., 2006), it would be plausible to speculate that although a smaller number of those cells arrive in the heart of infected mice, they may exhibit higher cytotoxicity. This would explain why the number of parasites in the cardiac tissue is not as great as one would expect. Additional studies are required in the quest to gain a greater insight on the immunobiological effects induced by Tc-TS proteins on T cells following *T. cruzi* infection.

The lack of methodological tools to study the biological roles of Tc-TS proteins over the course of *T. cruzi* infection, directs researchers toward alternative approaches for this technical deadlock. Therefore, the use of recombinant Tc-iTS and Tc-aTS, together or separately, presents a solution for studying the biological roles of Tc-TS proteins. Although there is no information regarding the expression levels of Tc-TS proteins, it would be plausible to imagine that following *T. cruzi* infection, numerous factors would contribute to both being expressed in different amounts. Over the last 10 years, studies demonstrated that when administered separately, both proteins may elicit similar biological effects, such as endothelial (Dias et al., 2008) and T cell activation (Todeschini et al., 2002a,b) and production of inflammatory signals (Ruiz Diaz et al., 2015). Nevertheless, there is no published work describing their combined effects. Our results demonstrated that separately, only Tc-aTS was able to modulate the expression of inflammatory signals by splenic T cells from *T. cruzi*-infected mice. Analysis of the peripheral blood parasitemia corroborated our previous findings (Freire-de-Lima et al., 2010), as well as the results described by Chuenkova and Pereira (1995), since only the administration of Tc-aTS was able to increase the number of blood trypomastigotes in *T. cruzi*-infected mice. However, when both proteins were injected in equal amounts, the effects triggered by Tc-aTS were significantly attenuated and/or abrogated.

The data contained in this paper presents, for the first time, evidence that Tc-iTS can assuage Tc-aTS effects in *T. cruzi*-acutely infected mice. Also, our results suggest that even though Tc-aTS and Tc-iTS present great structural similarity and compete for the same sialyl-glycan epitopes, they might present distinct immunomodulatory properties on host leukocytes following *T. cruzi* infection.

## Author Contributions

Conceived and designed the experiments: LF-d-L, CT, LM-P, and JP. Performed the experiments: LF-d-L, CT, LG, MN, LJ, LMF, KdC, and JL. Analyzed the data: LF-d-L, LG, MN, LJ, LMF, KdC, and JL. Contributed reagents/materials/analysis tools: CF-d-L, AM, CT, and MN. Wrote the paper: LF-d-L, LG, LMF, JP, and LM-P.

## Conflict of Interest Statement

The authors declare that the research was conducted in the absence of any commercial or financial relationships that could be construed as a potential conflict of interest.

## Supplementary Material

FIGURE S1 *Trypanosoma cruzi*-infected mice treated with Tc-aTS present higher amounts of parasite DNA in the cardiac tissue. BALB/c mice were infected with *T. cruzi* and treated or not with Tc-iTS or Tc-aTS. On 15th dpi, mice were euthanized, and the cardiac tissue processed for real time PCR analysis as described under “Materials and Methods.” Data are the mean ± SD of the parasite DNA in the cardiac tissue. (^∗^*p* ≤ 0.05 versus I and I-iTS groups), *n* = 4 animals per group.

FIGURE S2 Recombinant Tc-iTS decreases Tc-aTS virulence. Balb/c mice were infected and treated or not with Tc-iTS or Tc-aTS. In order to investigate if Tc-iTS can assuage the effect of Tc-aTS on blood peripheral parasitemia, *T. cruzi*-infected mice were injected with equal amounts of Tc-TS proteins, as well as an additional point where Tc-iTS amounts were thrice those of Tc-aTS. From the 6th to the 10th dpi, parasitemia was evaluated as described under the “Materials and Methods” section. The graph shows mean ± SD. [^∗^*p* ≤ 0.001 versus I group, ^∗∗^*p* ≤ 0.001 versus I-iTS group, ^+^*p* ≤ 0.001 versus I-aTS group, and ^++^*p* ≤ 0.001 versus I-iTS:I-aTS (1:1) group], *n* = 6 animals per group.
