# Supplementary figures and images for: Role of Inactive and Active Trypanosoma cruzi Trans-sialidases on T Cell Homing and Secretion of Inflammatory Cytokines
Source: Front Microbiol. 2017 Jul 11;8:1307. doi: 10.3389/fmicb.2017.01307 (PMC5504189; doi:10.3389/fmicb.2017.01307)

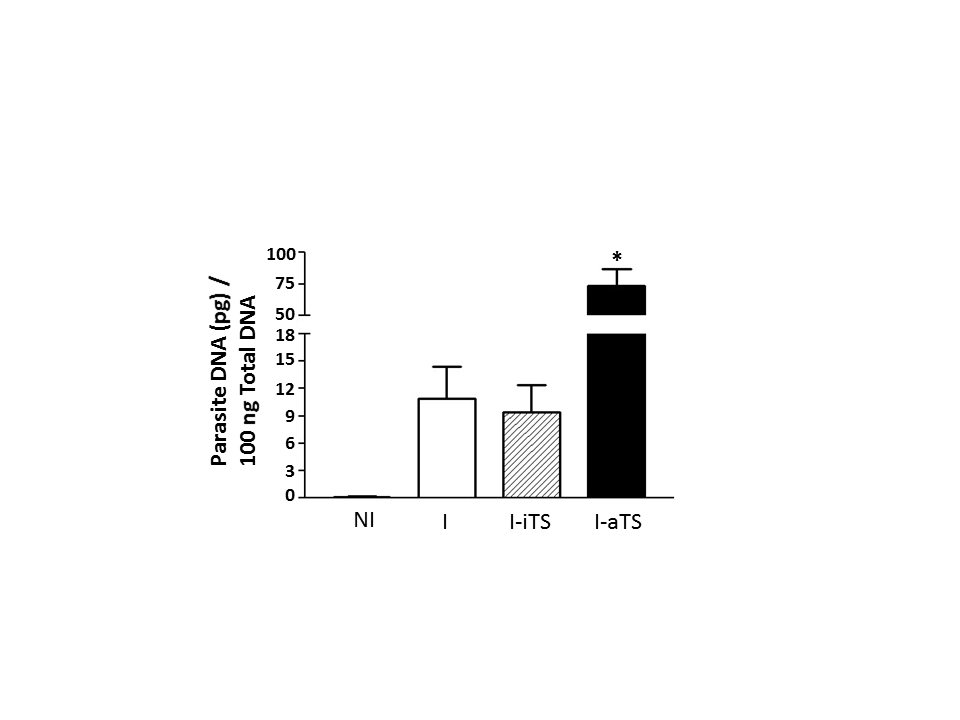

Supplement: FIGURE S1 — Trypanosoma cruzi-infected mice treated with Tc-aTS present higher amounts of parasite DNA in the cardiac tissue. BALB/c mice were infected with T. cruzi and treated or not with Tc-iTS or Tc-aTS. On 15th dpi, mice were euthanized, and the cardiac tissue processed for real time PCR analysis as described under “Materials and Methods.” Data are the mean ± SD of the parasite DNA in the cardiac tissue. (∗p ≤ 0.05 versus I and I-iTS groups), n = 4 animals per group. [file Image_1.TIF]

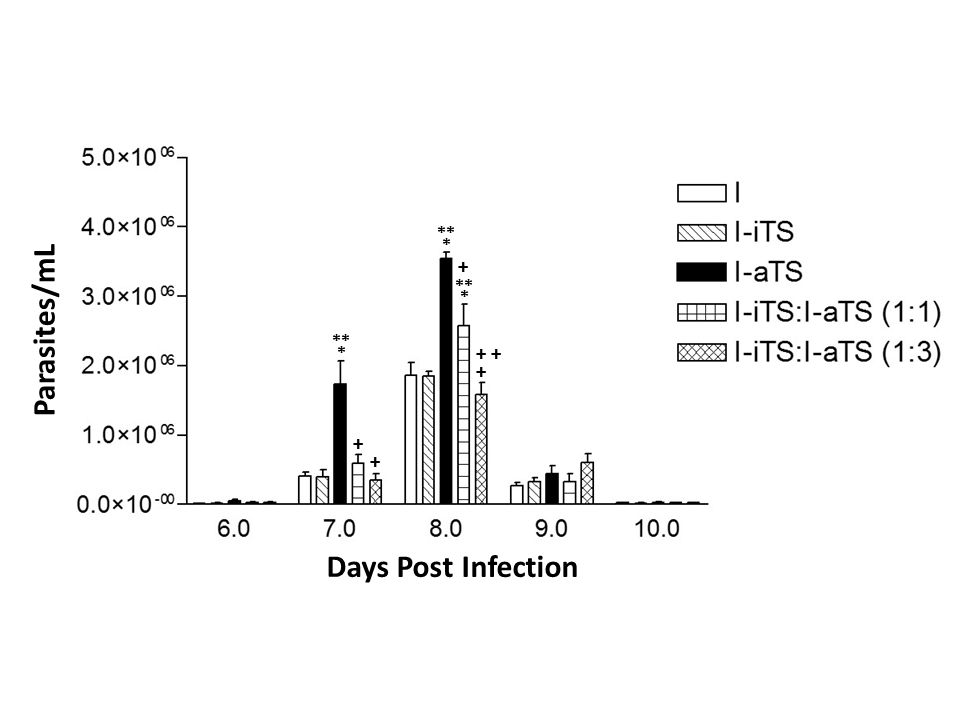

Supplement: FIGURE S2 — Recombinant Tc-iTS decreases Tc-aTS virulence. Balb/c mice were infected and treated or not with Tc-iTS or Tc-aTS. In order to investigate if Tc-iTS can assuage the effect of Tc-aTS on blood peripheral parasitemia, T. cruzi-infected mice were injected with equal amounts of Tc-TS proteins, as well as an additional point where Tc-iTS amounts were thrice those of Tc-aTS. From the 6th to the 10th dpi, parasitemia was evaluated as described under the “Materials and Methods” section. The graph shows mean ± SD. [∗p ≤ 0.001 versus I group, ∗∗p ≤ 0.001 versus I-iTS group, +p ≤ 0.001 versus I-aTS group, and ++p ≤ 0.001 versus I-iTS:I-aTS (1:1) group], n = 6 animals per group. [file Image_2.TIF]
